# Cancer immunotherapy with multiple tumor antigen activated autologous T cells in patients with HBV related hepatocellular carcinoma

**DOI:** 10.1186/2051-1426-2-S3-P245

**Published:** 2014-11-06

**Authors:** Yanyan Han, Ran Tao, Jin Huang, Yabing Guo, Dongyun Wu, Yujing Liao, Jin Li, Xiangjun Zhou, Jinlin Hou

**Affiliations:** 1 SYZ Cell Therapy Co., & Department of Infectious Diseases and Hepatology Unit, Nanfang Hospital, Peoples Republic of China; 2 SYZ Cell Therapy Co., Peoples Republic of China; 3 Department of Infectious Diseases and Hepatology Unit, Nanfang Hospital, Peoples Republic of China

## Background

Hepatocellular carcinoma (HCC) is one of the most common tumors in China, and frequently occurs in patients with chronic hepatitis B virus (HBV) infection. Although liver resection and other therapies may improve survival, HCC is rarely cured and with high risk of recurrence and metastasis. Here we present a practical adoptive immunotherapy named "Smart T" to prepare multiple tumor antigens activated T lymphocytes *ex vivo*, with a promising outcome in a preliminary clinical applications with HCC patients.

## Methods

Auotologous T cells from HCC patients were stimulated with mature DCs pulsed with multiple synthetic HCC-antigen peptides pool. The resulting T cells were infused into patients every 2-3 months with 5-10x10^7^cells/kg body weight for multiple cycles.

## Results

**Figure 1:**
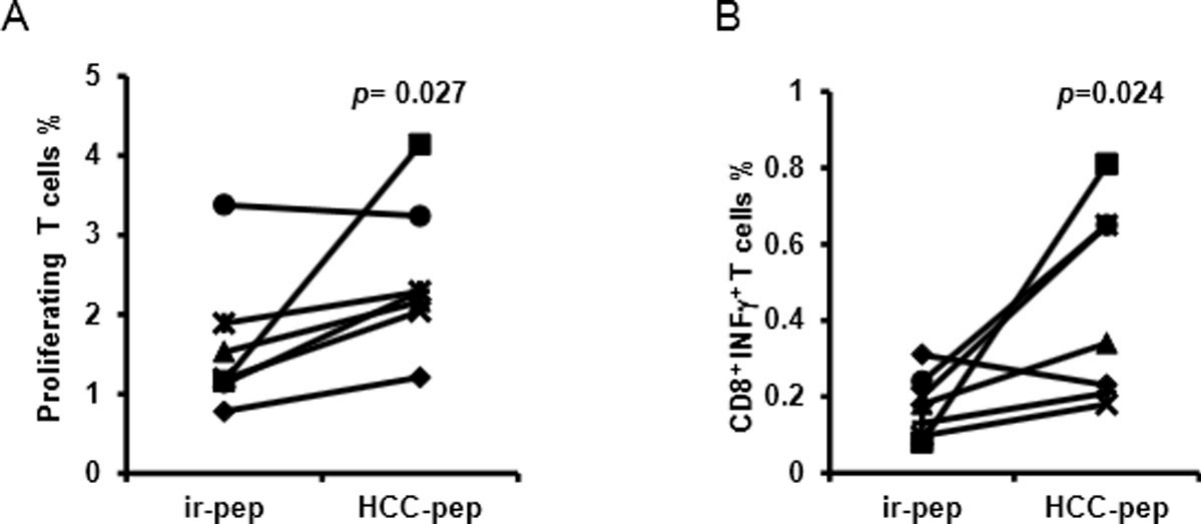


**Figure 2:**
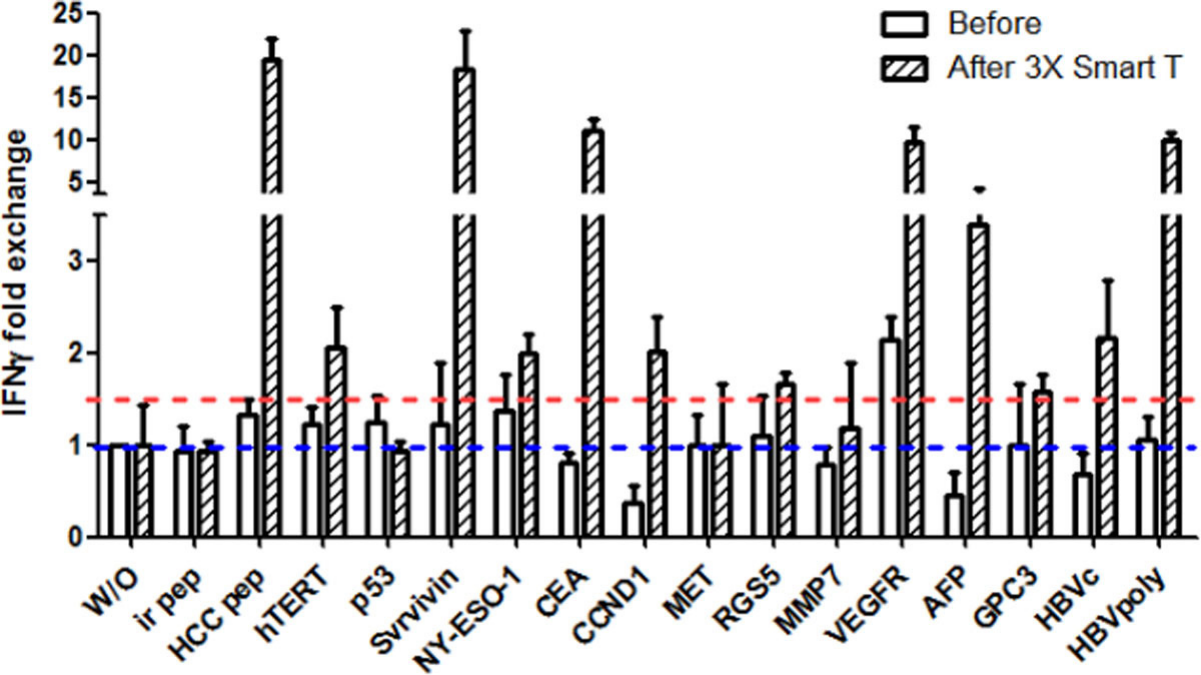


The resulting cells are mainly polyfunctional T cells (95% ± 1%) co-expressing INF-γ, TNFα and Granzyme B. The activated T cells generated from HLA-A2^+ ^patient exhibited greater cytotoxic activity to the HLA-A2^+ ^HCC cells than to the HLA-A2^- ^HCC cells. Up to date, 138 HCC patients have received "Smart T" treatments. No toxicity was observed. After 3 cycles of "Smart T" treatments, we have detected a significant increase of CD8^+^CD107a^+ ^T cells (p = 0.0082), effector T cells (p = 0.0013) and central memory T cells (p = 0.0038) in the patients' PBMCs as well as a significant decrease of regulatory T cells (p = 0.0015). Specific proliferation and IFN-γ production of T cells stimulated by HCC- antigens pool was also detected in the HCC patients' PBMCs as compared to irrelevant antigen stimulation (Figure 1). Moreover, the IFN-γ ELISPOT assay (Figure 2) demonstrated specific responses in patients against each kind of peptides, most of them responded to carcinoembryonic antigen (CEA), survivin, vascular endothelial growth factor receptor (VEGFR), alpha fetoprotein (AFP), glypican-3 (GPC3), HBV core antigen and HBV DNA polymerase. A retrospectively clinical analysis revealed that after multiple "Smart T" treatments, the recurrence and metastasis incidence in one year was low to 10% in the patients with B stage of HCC (n = 10), compared to B stage HCC patients without "Smart T" treatment (n = 14, recurrence and metastasis incidence: 71%, *p *= 0.005, analyzed by Fisher's exact 2-sided test).

## Conclusion

Our study, for the first time, demonstrates tumor antigens specific T cell responses can be robustly raised in HCC patients after Smart T treatment, and provides a safe treatment which may improve the immunologic function and clinical outcome of the HCC patients.

## Consent

Written informed consent was obtained from the patient for publication of this abstract and any accompanying images. A copy of the written consent is available for review by the Editor of this journal.

